# Predictive Value of Circulating Tumor Cells Detected by ISET^®^ in Patients with Non-Metastatic Prostate Cancer Undergoing Radical Prostatectomy

**DOI:** 10.3390/life12020165

**Published:** 2022-01-22

**Authors:** Laura Nalleli Garrido Castillo, Arnaud Mejean, Philippe Vielh, Julien Anract, Alessandra Decina, Bertrand Nalpas, Naoual Benali-Furet, Isabelle Desitter, Patrizia Paterlini-Bréchot

**Affiliations:** 1 Institut Necker Enfants Malades (INEM), INSERM U1151, Faculté de Médecine, Université de Paris, 75015 Paris, France; laura.garrido-castillo@inserm.fr (L.N.G.C.); julien.anract@aphp.fr (J.A.); 2 INSERM U807, Faculté de Médecine, Université de Paris, 75015 Paris, France; naoual.furet@bbox.fr; 3 Service d’Urologie, Hôpital Européen Georges Pompidou, 75015 Paris, France; arnaud.mejean@aphp.fr; 4 Medipath and American Hospital of Paris, 92200 Paris, France; ph.vielh@outlook.com; 5 Service d’Urologie, Hôpital Cochin, 75005 Paris, France; 6 Rarecells Diagnostics, 75280 Paris, France; alessandra.decina@rarecells.com (A.D.); isabelle.desitter@rarecells.com (I.D.); 7 Service d’addictologie, Université de Montpellier, 34090 Montpellier, France; bertrand.nalpas@inserm.fr; 8 Laboratoires de Biochimie Hôpital Necker-Enfants Malades, 75015 Paris, France

**Keywords:** Circulating Tumor Cell (CTC), prostate cancer (PCa), liquid biopsy, cancer recurrence

## Abstract

There is an unmet need for reliable biomarkers to predict prostate cancer recurrence after prostatectomy in order to better guide the choice of surgical treatment. We have evaluated the predictive value of the preoperative detection of Circulating Tumor Cells (CTC) for prostate cancer recurrence after surgery. A cohort of 108 patients with non-metastatic prostate adenocarcinoma undergoing radical prostatectomy was tested for the presence of CTC before prostatectomy using ISET^®^. Disease recurrence was assessed by the increase in serum PSA level after prostatectomy. The following factors were assessed for statistical association with prostate cancer recurrence: the presence of CTC, serum PSA, Gleason score, and pT stage using univariate and multivariate analyses, with a mean follow-up of 34.9 months. Prostate cancer recurrence was significantly associated with the presence of at least 1 CTC at the preoperative time point (*p* < 0.001; Predictive value = 0.83). Conversely, the absence of prostate cancer recurrence was significantly associated with the lack of CTC detection at diagnosis (Predictive value = 1). Our multivariate analysis shows that only CTC presence is an independent risk factor associated with prostate cancer recurrence after prostatectomy (*p* < 0.001). Our results suggest that CTC detection by ISET^®^ before surgery is an interesting candidate predictive marker for cancer recurrence in patients with non-metastatic PCa.

## 1. Introduction

According to GLOBOCAN 2020, prostate cancer (PCa) is the third cause of cancer-related death among men over 65 years. Most of the patients have an organ-confined tumor at diagnosis. Despite efforts to diagnose and treat it at early stages, biochemical recurrence (BCR) occurs in approximately 30% of patients after prostatectomy [1]. BCR is defined as a re-increase in serum PSA above >0.2 ng/mL after radical prostatectomy (RP) or <1 ng/mL after radiotherapy [2]. BCR could be due to incomplete surgical resection or small metastases in distant organs. However, BCR can occur in patients with cancer-free surgical margins, raising the hypothesis that Circulating Tumor Cells (CTC) could have been spread in the blood before prostatectomy and created micrometastases able to secrete PSA in the serum. CTC are metastatic precursors shed from primary tumors or metastasis into the bloodstream or lymphatic system [3]. Prediction of the risk of PCa recurrence after surgery is critical for the choice of surgical option versus non-surgical approaches. Given the known drawbacks and risks of prostatectomy (e.g., sexual impotence, urinary incontinence, etc.), new markers are needed to be able to predict when the surgical resection of the prostate is able to eradicate the PCa. Currently, a restricted number of tests are used as predictors of recurrence, such as preoperative serum PSA, patient age, biopsy-based Gleason score, and number of positive biopsy cores, all of them with limited accuracy [4,5].

Patients with intermediate or high-risk localized PCa undergo systematic staging imaging (computed tomography (CT), associated with bone scintigraphy or positron emission tomography (PET), or magnetic resonance imaging (MRI)) to detect locally advanced or metastatic diseases. Patients with locally advanced or metastatic diseases do not benefit from prostatectomy [6]. However, none of these imaging techniques is predictive such as CTC could do [7]. CTC could be a useful preoperative marker for cancer recurrence after surgery, and it could allow us to better select patients who will benefit from prostatectomy and help identify those patients who should be strictly monitored and treated with adjuvant therapies after surgery to avoid BCR.

The aim of this study was to assess, in a proof of principle approach, the potential clinical value of CTC as a pre-surgery marker to help identify patients at risk of developing recurrence after surgery, thus helping patients’ stratification for surgical treatment and adjuvant treatment after surgery.

Several strategies have been developed to isolate and detect CTC either by marker-dependent or marker–independent approaches. Marker-dependent methods may lead to selection biases, false positive and false negative results, due to CTC heterogeneity (cells with either epithelial or mesenchymal phenotype or undergoing a transformation from an epithelial to a mesenchymal phenotype). CellSearch^®^ system, which is a marker-dependent method, is currently the FDA-cleared method for CTC enumeration (CTC cut off of ≥5 CTCs in 7.5 mL blood) to predict progression-free survival and overall survival in patients with metastatic PCa. Despite this, CellSearch’s clinical value in non-metastatic PCa is debated [8]. In this work, we used the Isolation by Size of Tumor cells (ISET^®^) technology (Rarecells Diagnostics, Paris, France), a label-free approach, to assess whether the preoperative presence of CTC in the blood is associated with disease recurrence after surgery in patients diagnosed with non-metastatic prostate cancer.

## 2. Patients and Methods

### 2.1. Study Patient Cohort

**Figure 1:**
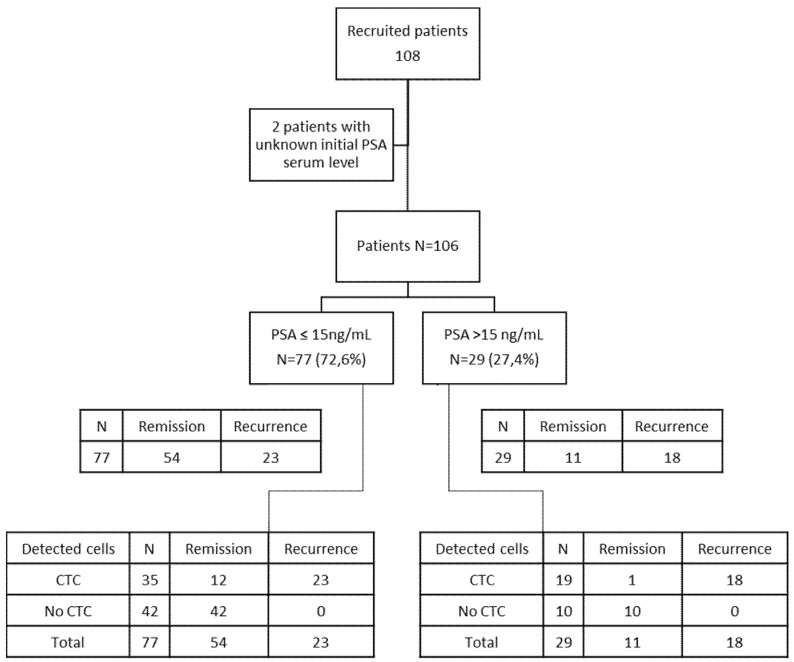
Consort flow diagram showing the CTC results and baseline PSA values in 106 out of 108 patients.

A total of 108 patients with non-metastatic PCa undergoing radical prostatectomy were consecutively included in this study and recruited at the Necker and HEGP hospitals in Paris. See Table 1 for baseline patients’ characteristics, including the administered postoperative treatments, and Figure 1 for the consort flow diagram. All patients were tested for the presence of CTC using ISET^®^ before surgery and before, or at least three weeks after, any medical invasive procedure (digital rectal examination, transrectal ultrasound, biopsy, etc.) which could iatrogenically spread CTC in blood. All patients had been diagnosed with PCa by biopsy. Inclusion criteria were: patients with newly diagnosed, untreated prostate cancer undergoing prostatectomy; not having been diagnosed with a different tumor before the inclusion; agreeing to participate in the study; having the French Social Security affiliation.

Seventy-seven patients (72.6%) had PSA ≥15 ng/mL at diagnosis. The majority of patients had a Gleason score of 7 or more at the biopsy (58/107 = 54.2%). The majority of patients had pT stage T2b or higher (87/108 = 80.6%) at the pathologic analysis after prostatectomy (Table 1).

The majority of patients, 92 (85.2%), were treated by radiotherapy plus chemotherapy plus androgen-deprivation therapy (ADT), while the rest of the patients received either ADT, radiotherapy or no treatment (Table 1).

The data about the prostate volume could not be collected.

Disease recurrence was defined by an increase in serum PSA level post-surgery to 0.2 ng/mL or higher in two independent tests, defined as biochemical recurrence (BCR). Patients with BCR could also be studied, in a case-by-case manner, with imaging (CT, or PET, or MRI). The median follow-up after prostatectomy was 34.9 months (range 6.3–75 months).

Fifty healthy controls were included in this study: men aged 55 to 75 yrs, without known pathology, including without BPH (Benign Prostatic Hyperplasia), agreeing to participate in the study, having the French Social Security affiliation.

Men with BPH were excluded because of the frequent association of BPH with prostate cancer.

### 2.2. Circulating Tumor Cells Analysis

To evaluate the presence of CTC, peripheral blood samples (6 mL) were collected in EDTA tubes before prostatectomy far (see above) from any possible iatrogenic cause of CTC spreading. Blood was filtered using ISET^®^ as described previously [9]. Briefly, blood samples were diluted with ISET^®^ Buffer 1:10 and, after incubation (10 min), were filtered using the ISET^®^ platform. ISET^®^ membranes were then washed (PBS), dried, and stored at −20 °C. Hematoxylin and eosin staining was done directly on ISET^®^ membranes for cytomorphologic analysis. The blood (6 mL) from healthy controls was filtered and analyzed in the same manner.

The isolated circulating rare cells were analyzed to identify cells with fully malignant characteristics allowing us to diagnose them as Circulating Tumor Cells (CTC). The following criteria were used to characterize cell malignancy: nucleus larger than 3 calibrated pore size of the membrane (>24 μm), irregular nuclear borders, anisonucleosis, nuclear hyperchromatism, high nucleocytoplasmic ratio (ratio > 0.5), size and number of nucleoli, and presence of tridimensional sheets. CTC was then defined by the presence of at least three of these criteria [10]. Pathologists (PV and NB (acknowledged)) agreed on these criteria and did not report any discordant cell diagnosis).

### 2.3. Statistical Analysis

Continuous variables were compared using Student’s test or a non-parametric (Mann–Whitney) test when their distribution was skewed. Categorical variables were compared using Chi-square or the Fisher’s exact test. Multivariate analysis was done using the logistic regression method. The association of serum PSA, Gleason scores, and CTC with cancer relapse was evaluated by univariate and multivariate analysis. The cumulative survival rates were analyzed using the Kaplan–Meier method, and curves were compared using the log-rank test. All analyses were conducted in R (R Development Core Team, 2021). A *p*-value < 0.05 was considered statistically significant for all statistical analyses.

## 3. Results

Based on cytopathological analysis of the cells enriched by ISET^®^, we detected CTC in 55 out of 108 (50.9%) patients, and in 0 out of 50 healthy controls. The cytopathological analysis of cells isolated by ISET^®^ allowed us to categorize patients into three groups based on the number of detected CTC (0, 1 to 3, and more than 3 cancer cells) per 6 mL of blood. Table 2 shows the outcome of remission or recurrence according to the three different groups. The average CTC count in patients was 1.6 cells per 6 mL of blood, ranging from 1 to 14.

**Figure 2:**
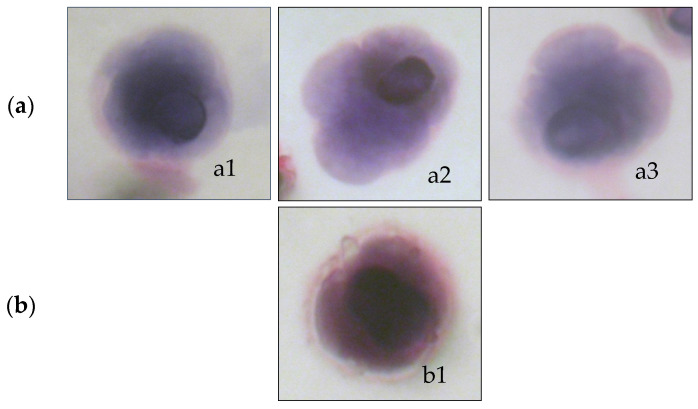
Cytomorphological analysis of cells isolated by ISET^®^: (**a**) a1, a2, and a3 illustrate circulating tumor cells (CTC) with full characteristics of tumor cells, and (**b**) b1 illustrates a circulating fragile tumor cell (CFTC) with a tumor-like nucleus and damaged cytoplasm.

Cytopathologists noted the presence of cells having a tumor-like nucleus, damaged cytoplasm, or often incomplete criteria of malignancy. These cells, collectively named CFTC (Circulating Fragile Tumor Cells), were identified and counted. However, classical cytopathological criteria do not take them into account. Figure 2 shows an example of CTC and a CFTC.

A survival without recurrence curve analysis was performed to look at the correlation of CTC numbers and PCa recurrence. Both subgroups with CTC (1 to 3 and >3 CTC per 6 mL of blood) were associated with a significantly higher PCa recurrence than CTC free patients (*p* < 0.0001).

**Figure 3:**
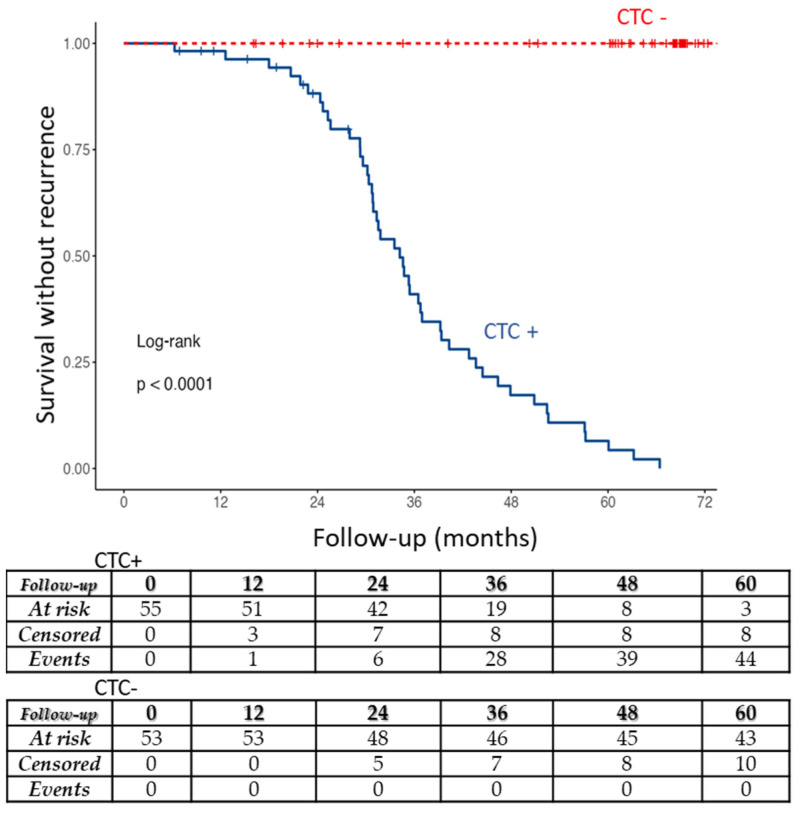
Survival without recurrence curve. Subgroups of patients: with CTC (CTC+), and without CTC (CTC-). The table at the bottom shows CTC+ and CTC- patients at risk of recurrence (At risk), censored patients and cases of PCa recurrence (Events) at 0, 12, 24, 36, 48, and 60 months during follow-up after prostatectomy. Risk of recurrence was highly significantly associated with CTC positivity: *p* < 0.0001. CFTC were not taken into account for this analysis.

Figure 3 shows the survival without recurrence curve analysis depicting the correlation of CTC positivity with PCa recurrence after surgery. The correlation is statistically highly significant (*p* < 0.0001). Figure 3 (bottom) also shows the number of censored patients and recurrent cases.

PCa recurrence was thus significantly associated with the presence of at least 1 CTC detected before surgery (*p* < 0.001; positive predictive value = 0.83, 46/55), and the absence of recurrence was significantly associated with the lack of CTC detection (negative predictive value = 1, 53/53).

Concerning CFTC, 89 out of 108 (75.9%) patients were positive before prostatectomy. The average CFTC count in these positive individuals was 2.1, ranging from 1 to 14. Both CFTC and CTC were detected in 52 patients and only CFTC in 37 patients. Their presence was often detected along with CTC presence as only 3 patients had CTC only (without CFTC). An absence of CFTC was found in 19 patients. The predictive value for recurrence of CFTC is 0.48, far less than the value of CTC (0.83), supporting the view that they are probably dying cells not able to generate metastases, consistent with the cytopathological view.

**Figure 4:**
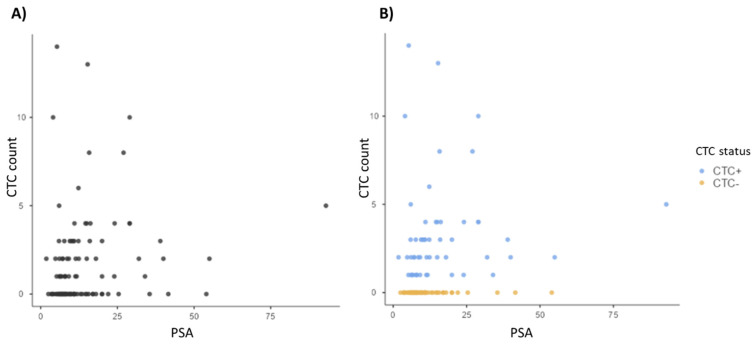
Scatterplots showing (**A**) no correlation (*p* = 0.099) between serum PSA level at baseline (ng/mL) and the CTC count (N° of CTC per 6 mL of blood) in all patients and, (**B**) no correlation (*p* = 0.553) between serum PSA level at baseline (ng/mL) and CTC count (N° of CTC per 6 mL of blood) in CTC positive (blue dots) and CTC negative (orange dots) patients.

We did not find a correlation between serum PSA at baseline and the presence or absence of CTC (*p* = 0.079). We also did not find a correlation between PSA and CTC count, taking into account all patients together (*p* = 0.099) or the two subpopulations of patients (CTC+ and CTC−) (*p* = 0.553). Figure 4 shows the scatterplot of serum PSA and CTC count (patients were divided into two categories CTC positive and CTC negative).

We observed that PSA level ≥ 15 ng/mL is significantly associated with PCa recurrence (*p* = 0.002), while Gleason score ≥ 7 was not (*p* = 0.27).

We studied the correlation between the pT staging and the presence of CTC. Patients with T2a tumors had a lower CTC positivity rate (4 CTC positive patients out of 21 T2a) (*p* = 0.013). Patients with T2b tumors or higher stage had a significantly higher CTC positivity rate (51 CTC positive patients out of 87 (*p* < 0.001)). No significant difference in CTC frequency was found among patients with T2b, T3a, and T3b stages (T2b-59.1%, T3a-60.9%, T3b-55.0%).

To note, the CTC predictive value for the diagnosis of a tumor stage equal or greater than T2b is 0.93. In fact, out of 55 CTC positive patients, 51 were classified as stage T2b or higher. We also found that patients with tumor stage equal to or higher than T2b had a significantly higher frequency of recurrence (*p* = 0.038). 

Table 3 shows the results of the statistical univariate and multivariate analysis for the association of different parameters with PCa recurrence. In the multivariate analysis, we studied the three parameters available before prostatectomy (CTC, PSA value, and Gleason score) and the pT stage obtained from the pathological analysis of the surgical sample. Only preoperative CTC detection was found to be an independent risk factor associated with PCa recurrence (*p* < 0.001).

## 4. Discussion

In this study, we evaluated the clinical impact of CTC detected by ISET^®^ in patients with PCa undergoing prostatectomy. It is worth pointing out that ISET^®^ was used to assess the cytomorphological characteristics of CTC and count them using a universally recognized diagnostic approach. Few studies evaluated the clinical impact of CTC in patients with non-metastatic PCa [8,11]. So far, the relationship between CTC before prostatectomy and PCa recurrence has not been reliably estimated due to the rarity of CTC in the blood at early cancer stages, the variable sensitivity of the methods used, and the lack of diagnostic approaches used to identify CTC. Thus, we thought of applying ISET^®^, known for its extremely high sensitivity [12] and diagnostic approach to counting the CTC, to this field.

Our results show that ISET^®^ could find CTC in 55 (50.9%) of the patients before prostatectomy, showing that the ISET^®^ technology allows CTC enrichment at early PCa stages. Survival without recurrence curves showed that the presence of CTC, without difference between 1 to 3 CTC and more than 3 CTC per 6 mL of blood, was highly significantly associated with the risk of recurrence (*p* < 0.001).

By studying, in a multivariate analysis, all parameters which are available before prostatectomy (CTC, PSA, and Gleason score) and the pT stage, we have observed a strong correlation between the presence of CTC and cancer recurrence, with CTC being an independent risk factor significantly associated (*p* < 0.001) with PCa recurrence after prostatectomy. PCa recurrence was thus significantly associated with the presence of at least 1 CTC detected before surgery (positive predictive value = 0.83), and the absence of recurrence was significantly associated with the lack of CTC detection (negative predictive value = 1).

These very interesting results can presumably be explained by the fact that we used a marker-independent method to extract CTC from blood with high sensitivity, proven to detect CTC at early stages in prostate and other cancer types [7,13,14] and a diagnostic method to diagnose CTC.

The use of CTC as a biomarker for localized and locally advanced PCa has been limited due to technical challenges related to the CTC rarity and heterogeneity. Table 4 shows previous studies analyzing CTC presence and number in patients with non-metastatic PCa and the methods used. CTC isolation methods based on a surface marker, mainly CellSearch, are less sensitive and have lower CTC detection rates than marker-independent approaches. As we can see in Table 4, 9 out of 15 studies used a marker-dependent approach and 8 of 9 used CellSearch. A total of 4 of the 9 studies included a follow-up after surgery, and none reported a statistically significant correlation of CTC detection with PCa recurrence. However, as mentioned, marker dependent methods show a lower rate of CTC positive patients.

As a matter of fact, several publications have shown the superior sensitivity of ISET^®^ (marker independent) as compared with CellSearch (marker dependent) when applied to prostate cancer and other types of cancer [15,16,17,18,19,20,21]. Detection rates in blood samples were: 50% vs 39% [16]; 75% vs 32% [17]; 93% vs 40% [19]; and 80% vs 23% [20], using ISET^®^ and CellSearch, respectively.

Among 6 studies [22,23,24,25,26,27] using marker-dependent CTC isolation methods and reporting a CTC detection rate equal or higher than 50%, 3 did not show correlation between CTC and clinical variables, or have a follow-up. Among the remaining three studies with follow-up (from 14.2 months to 5 years), Todenhöfer et al. [22] did not study the correlation of CTC with PCa recurrence, Salami et al. [23] did not find a correlation between baseline CTC and BCR (*p* = 0.10), and Murray et al. [24] found a significant correlation of BCR with PSA, Gleason score, T3 stage, CTC positivity, and higher CTC counts (*p* < 0.05). Thus, our study confirms the results obtained by Murray et al. Furthermore, our study found a stronger association of CTC presence with BCR (*p* < 0.001), which is probably related to the specificity of the cytopathological method used to identify the CTC.

Consistently with previous studies which have reported that CTC is not detectable in blood samples from healthy donors using the ISET^®^ approach [7,9,10,37,38,39,40,41,42,43,44,45,46,47,48], we did not detect CTC in 50 healthy volunteers.

It is important to note that we did not include patients with BPH in the control group because BPH and prostate cancer are considered to be linked by common physiopathological factors [49] and frequently coexist in men aged < 65 years, as was shown in studies using transurethral prostatic resection [50].

Cytopathology is known to be extremely specific. As a matter of fact, to this date, 539 healthy volunteers and 200 patients with benign diseases have been tested by ISET^®^ in 16 studies, setting the specificity of ISET blood cytopathology at 98.6% (10/739) [7,9,10,37,38,39,40,41,42,43,44,45,46,47,48,51]. However, its sensitivity is hard to assess, especially in the setting of circulating tumor cells analysis. A blind study that analyzed CTC in renal cell carcinomas carrying VHL mutation found the VHL mutation in all the CTCs isolated from the blood using ISET^®^. Results revealed that all the cells diagnosed as CTC by the cytopathological analysis carried the VHL mutation detected in the corresponding tumor tissue. Conversely, 104 out of 125 cells, defined as having uncertain malignant features according to pathological criteria, were, in fact, CTCs as they carried the identical VHL mutation also identified in the corresponding tumor tissue [52]. According to this study, the specificity of cytopathology was 100%, while the sensitivity was 72%.

This type of study is not possible yet in patients with prostate cancer due to the lack of suitable molecular markers. Some of them have been described as predictors of therapy response or ways of helping to guide therapies such as TMPRSS2-ERG fusion, PTEN status, presence of AR-V7 splice variant, mutations in DNA-repair genes such as BRCA2/1, etc. [53] in metastatic prostate cancer patients. However, we still do not know the genetic markers of prostate cancer that are present in all the tumor cells from the different prostate tumor types. Thus, the same type of comparative molecular versus morphological analysis that we have done in patients with kidney cancer cannot be performed in patients with prostate cancer. We are confident, anyway, that these diagnostic molecular markers will emerge in the near future.

In this study, we did not perform genetic analysis of patient DNA, nor of tumor or CTC DNA. Alterations in DNA repair pathways, such as single-nucleotide polymorphisms (SNPs) or germline mutations, are associated with PCa development, aggressiveness, and progression. Unfortunately, the rate of patients harboring these alterations at early-stage PCa is low (7–12%) [54].

Invasive tests based on genomic classifiers from tumor tissue, such as Oncotype DX Genomic Prostate Score and Decipher, are now commercially available as nomograms guiding PCa treatments and predicting metastasis and cancer mortality. Reports using those tests for predicting BCR showed that higher scores were independently associated with BCR (HR/5 units 1.14, 95% CI 1.03–1.26, *p* = 0.01) [55] with an accuracy of around 80% [56]. However, these tests require the DNA from the tumor tissue and cannot be used non-invasively and pre-surgery.

CTC characterization or subtyping by different biomarkers might help the applications of CTC in routine PCa management. Some cell markers such as EGFR, PSMA, PSA, AR [57], CD133 (stem cell marker), and E-cadherin (EMT) [31] have been used without a clear association to predict cancer outcomes. Other markers such as vimentin, PSA, and PSMA can be used for CTC characterization. However, if the expression of these markers is low or barely detectable, the CTC characterization fail. It is well known that PSA expression is specific to prostate cells but not of prostate tumor cells, and PSMA, as well as PSA, is not expressed in all prostate tumor cells. In our study, we did not perform immunolabelling of cells isolated by ISET^®^, as labelling may hinder the cytopathological characteristics which have to be examined carefully by the cytopathologist to diagnose the presence of CTC. Our results show that, in our study targeting patients with newly diagnosed PCa, CTC detection by cytopathology without any other cell characterization is able to identify patients at higher risk of recurrence after prostatectomy.

An interesting point is the observation of CFTC in 75.9% of patients. We may hypothesize that the CFTCs we observed are CTCs detached from the tumor, dying because of anoikis, i.e., programmed cell death that occurs in cells upon loss of attachment to the surrounding extracellular matrix and neighboring cells. Thus, the nuclear characteristics remain “tumor-like” but the cell morphology is rapidly affected. In general, cytopathologists do not take into consideration cells that do not have a fully visible cytoplasm. However, it is natural to speculate that those possibly dying tumor cells do not have a real clinical impact on the disease outcome because of their presumed lack of viability. CFTC could derive from damages related to mechanical stress and cell–cell interactions [58]. Moreover, technical factors could have an impact on cellular morphology. Thus, more exhaustive studies have to be carried out in order to clarify the origin of the CFTCs and their significance. For now, we just want to attract attention to this finding hoping that more studies will be planned in the future targeting cells previously not described by the pathologists because they lack cellular integrity.

To conclude, our results show that CTC detection by ISET^®^ before prostatectomy could be a reliable biomarker for PCa recurrence, with better predictive value than serum PSA before surgery. Studies of larger cohorts of patients with localized prostate cancer tested before prostatectomy are needed to further validate our findings.

## Figures and Tables

**Table 1:** Patient clinical and pathological baseline characteristics.

Clinical Parameter		Number (%) or Median (Range)
Total patients		108 (100%)
Age (yrs)		65.1 (±8.6)
Preoperative serum PSA (ng/mL)		
Mean		13.92
Median		10.0
Range		1.83–93.00
<15 ng/mL		77 (77/106 = 72.6%)
≥15 ng/mL		29 (29/106 = 27.4%)
Unknown		2 (2/108 = 1.9%)
**Gleason Score**		
≤6		49 (49/107 = 45.8%)
7		48 (48/107 = 44.9%)
≥8		10 (10/107 = 9.3%)
Unknown		1 (1/108 = 0.9%)
**Pathological Stage—pT Staging**		
*T*	*T2a*	21 (19.4%)
	*T2b*	44 (40.7%)
	*T3a*	23 (21.3%)
	*T3b*	20 (18.5%)
*N*	*N0*	103 (95.4%)
	*N1*	5 (4.6%)
*M*	*M0*	107 (99.1%)
	*M1*	1 (0.9%)
**Initial treatment**		
Radical prostatectomy		108 (100%)
**Postoperatory Treatments**		
Radiotherapy + chemotherapy + ADT *		74 (68.5%)
Hormonal therapy (ADT)		10 (9.3%)
Radiotherapy		8 (7.4%)
None		16 (14.8%)

* ADT: Androgen-deprivation therapy.

**Table 2:** CTC remission and recurrence according to CTC count in the 108 patients undergoing prostatectomy.

CTC Count	N	Remission	Recurrence
No CTC	53	53	0
1–3 CTC	39	7	32
>3 CTC	16	0	16

**Table 3:** Statistical association of different parameters and PCa recurrence.

Prognostic Factor	*p* Value
Univariate analysis	
Gleason score 7/>7	0.27
PSA, >15 ng/mL	**=0.002**
Presence of CTC	**<0.001** (predictive value = 0.83)
CTC positive patients in treated group	**<0.001**
CTC positive patients in non-treated group	0.007
≥T2b tumors (pT staging)	0.038
Multivariate analysis	
CTC presence	**<0.001**
PSA	0.497
Gleason score	0.172
pT stage	0.177

**Notes:** significant values are marked in bold. In multivariate analysis (serum PSA, Gleason, CTC, and pT stage) only preoperative CTC detection was an independent risk factor associated with PCa recurrence (*p* < 0.001).

**Table 4:** Published studies assessing CTC counts in localized PCa.

Study	No. Patients	pT Stage	CTC Detection Method	Cutoff	CTC+ Patients (%)	Blood Sample Size	Results
**(Davis et al., 2008)** [28]	97	78 T2 19 T3	CellSearch	≥1 CTC/22.5 mL	20/97 (21%)	30 mL	No correlation between the number of CTC and tumor volume, pathological stage, and Gleason score.
**(Maestro LM et al., 2009)** [29]	24	Uninformed	CellSearch	≥2 CTC/7.5 mL	4 (14%)	10 mL	No correlation between CTC presence and tumor stage.
**(Thalgott et al., 2015)** [30]	20	locally advanced high risk	CellSearch	≥1 CTC	1 (5%)	7.5 mL before neoadjuva therapy and RP	No difference in patients CTC counts compared to controls. Follow-up 8–16 weeks following RP.
**(Kolostova et al., 2014)** [31]	55	45 T2 10 T3	MetaCell^®^ filtration	≥1 CTC	28 (52%)	8 mL	No correlation found with Gleason score or tumor stage.
**(Shao et al., 2014)** [25]	40	26 T2 13 T3 1 Tx	Near-infrared dyes	≥1 CTC	39 (97.5%)	7.5 mL	No correlation found with Gleason score, tumor stage, or PSA level.
**(Pal et al., 2015)** [32]	35	32 T1-T2, 3 T3	Ficoll- CellSearch	≥1 CTC	16 (49%)	30 mL	No association with clinical parameters. Median follow-up 510 days.
**(Murray et al., 2016)** [24]	269	Unknown	differential centrifugation + ICC	≥1 CTC	211 (79%)	8 mL	BCR was associated with PSA, Gleason score, T3 disease, CTC positivity, and higher CTC counts (*p* < 0.05). Median follow-up 5 years.
**(Kuske et al., 2016)** [26]	86	37 T1 45 T2 4 T3	CellSearch EPISPOT CellCollector	≥1 CTC	−37% CellSearch −54.9% CellCollect −58.7% EPISPOT	−7.5 mL-Directly from the vein −13–15 mL	CTC detected by EPISPOT correlated with tumor stage.
**(Todenhöfer et al., 2016)** [22]	50	37 T2 13 T3	Microfluidic device	≥1 CTC/2 mL	25 (50%)	2 mL	No correlation found with Gleason score, tumor stage, or PSA level. PCa recurrence was not studied. Median follow-up 48 months.
**(Tsumura et al., 2017)** [33]	59	26 T1c–T2a, 15 T2b–c, 17 T3, 1 T4	CellSearch	≥1 CTC/7.5 mL	0% (0/59) before and 11.8% (7/59) after surgery	10 mL	No correlation found with Gleason score, tumor stage, or PSA level. Median follow-up 18 months.
**(Puche-Sanz et al., 2017)** [34]	86	Unknown	Immune-magnetic	≥1 CTC/10 mL	16 (18.6%)	10 mL	No correlation with CTC counts. However, AR expression in tumor tissue correlated with CTC presence.
**(Salami et al., 2019)** [23]	26	2 pT2 15 pT3a9 pT3b	Epic Sciences	≥1	19 (73%)	10 mL	Metastasis (*p* = 0.03) was associated with baseline CTC detection while BCR (*p* = 0.10) was not. Median follow-up 14.2 months.
**(Liu et al., 2020)** [27]	80	5 T1c 37 T2a 11 T2b 23 T2c 4 T3a	CanPatrolTM	≥1 CTC/5 mL	44 (55%)	5 mL, before surgery	PSA levels and Gleason score had no correlation with CTC counts.
**(Zapatero et al., 2020)** [35]	65	1 T1 17 T2 47 T3	CellSearch	≥1 CTC	65 (7.5%) before treatment	7.5 mL	CTC status was not significantly associated with any clinical or pathologic factors. Detection of CTCs was not significantly associated with overall survival.
**(Knipper et al., 2021)** [36]	20	8 pT2 4 pT3a 7 pT3b 1 unknown	CellSearch	2–3 CTCs/7.5 mL	3 (15%)	7.5 mL	CTC-positive correlated with BCR-free survival (BFS). Median follow-up of 10.1 months.
